# Synthesis and Crosslinking of Polyether-Based Main Chain Benzoxazine Polymers and Their Gas Separation Performance

**DOI:** 10.3390/polym10020221

**Published:** 2018-02-23

**Authors:** Muntazim Munir Khan, Karabi Halder, Sergey Shishatskiy, Volkan Filiz

**Affiliations:** Helmholtz-Zentrum Geesthacht, Institute of Polymer Research, Max-Planck-Strasse 1, 21502 Geesthacht, Germany; muntazim.khan@hzg.de (M.M.K.); karabi.halder@hzg.de (K.H.); sergey.shishatskiy@hzg.de (S.S.)

**Keywords:** benzoxazine, polycondensation, membranes, gas transport properties, Jeffamine^®^

## Abstract

The poly(ethylene glycol)-based benzoxazine polymers were synthesized via a polycondensation reaction between Bisphenol-A, paraformaldehyde, and poly(ether diamine)/(Jeffamine^®^). The structures of the polymers were confirmed by proton nuclear magnetic resonance spectroscopy (^1^H-NMR), indicating the presence of a cyclic benzoxazine ring. The polymer solutions were casted on the glass plate and cross-linked via thermal treatment to produce tough and flexible films without using any external additives. Thermal properties and the crosslinking behaviour of these polymers were studied by thermogravimetric analysis (TGA) and differential scanning calorimetry (DSC). Single gas (H_2_, O_2_, N_2_, CO_2_, and CH_4_) transport properties of the crosslinked polymeric membranes were measured by the time-lag method. The crosslinked PEG-based polybenzoxazine membranes show improved selectivities for CO_2_/N_2_ and CO_2_/CH_4_ gas pairs. The good separation selectivities of these PEG-based polybenzoxazine materials suggest their utility as efficient thin film composite membranes for gas and liquid membrane separation technology.

## 1. Introduction

Membrane-based gas separation has attracted significant interest in the industrial sector due to its advantages over conventional separation methods. Polymeric materials are promising candidates for fabricating membranes due to their high competitiveness in performance and cost efficiency [1]. Although a vast number of materials have been reported, only a few polymeric materials have been used for industrial membrane-based separations (e.g., O_2_/N_2_, CO_2_/N_2_, CO_2_/CH_4_) [2]. The accomplishment of high-efficiency gas separation requires high performance membranes that possess both good permeability and permselectivity. However, there exists a trade-off between gas permeability and permselectivity, described by the Robeson upper-bound relationship [3]. 

Poly(ethylene glycol) (PEG) containing polymers have been extensively studied as membrane materials for CO_2_ separation from permanent gases. CO_2_ was highly soluble in PEG due to the quadrupole moment resulting in favourable intermolecular interactions with polar (ether) groups in polymers, where the diffusion of CO_2_ and other gases was relatively high and did not influence the selectivity because of the PEGs’ rubbery nature, leading to enhanced gas permeability and solubility selectivity [4,5,6]. In addition, hetero-aromatic polymers like polyimides and polyamides show specific physicochemical properties, such as a high value of gas pair selectivity (e.g., CO_2_/N_2_ or CO_2_/CH_4_) in gas separation, which make them promising materials for membrane technology applications [7,8,9]. Likely, oxazine, which is also a member of N-containing organic heterocyclic, is a favourable structure in a polymer membrane for CO_2_ separation. A common type of oxazine ring is 1,3-benzoxazine, which receives considerable attention in academia and the chemical industry. Benzoxazine polymerization has many distinct properties such as negligible volume shrinkage upon curing, does not require any harsh acid catalyst or additives for curing, high thermal stability, low water absorption, and high char-yield of the cured products [10,11,12,13]. Additionally, benzoxazines also have immense molecular design flexibility, which allows them to be tailored to a particular application simply by substituting different functionalities into the primary amine or phenolic reactants [14]. An area of benzoxazine research has been developed where polymeric benzoxazines were synthesized by replacing the starting monofunctional amines reactant (or monofunctional phenols) of classical monomeric benzoxazines with difunctional phenols (or difunctional amines) [15,16,17]. The resulting polymer with repeating benzoxazine units in the main chain is formed. At this point, the benzoxazine acts like an ordinary thermoplastic, which has good solubility, processability, and film formation properties. Upon curing at an elevated temperature, this thermoplastic polymer can be crosslinked via thermally activated ring-opening polymerization (ROP) of the oxazine ring in the main polymer chain. Thus, this thermoplastic/thermosetting crossover molecule gives the advantage of thermoplastic processability with thermosetting polymer characteristics. Good dimensional stability, creep resistance, and great thermal and chemical resistance were some of the common advantages of thermosetting polymers that were observed in cross-linked mainchain-type polybenzoxazines [18,19].

In this study, we report on the preparation of the PEG-based polybenzoxazine from Bisphenol A, aliphatic poly(ether diamine), in the presence of paraformaldehyde. The polymer structure was cured via thermally activated ring-opening polymerization (ROP). The crosslinked films were characterized by differential scanning calorimetry (DSC), thermogravimetric analysis (TGA), and gas-separation techniques.

## 2. Materials and Methods

### 2.1. Materials

Poly(ether diamine)s or Jeffamine^®^ of different molecular weights (95%) were kindly supplied by Huntsman. The chemical structure, composition, and molecular weight of Jeffamine^®^ are shown in Table 1.

Bisphenol-A (>99%), paraformaldehyde (96%) were used as purchased from Aldrich (St. Louis, MO, USA). Toluene (~99%) and hexane (~99%) were used as purchased from Merck (Kenilworth, NJ, USA).

### 2.2. Synthesis of PEG-Based Main Chain Benzoxazine Polymer

**Scheme 1:**
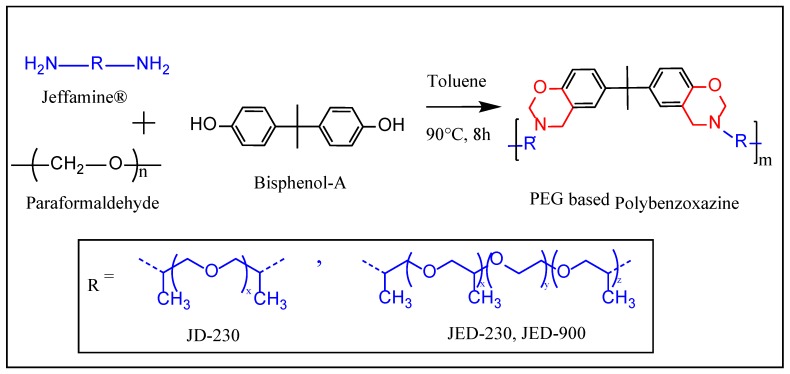
Synthesis of PEG-based main-chain polybenzoxazine.

All polymer synthesis followed the same procedure: an equimolar mixture of Jeffamine^®^ (1 mol) and Bisphenol-A (1 mol), and paraformaldehyde (4 mol) in toluene (20% solid content) was stirred with magnetic stirring at 90 °C for 8 h, according to Scheme 1. 

The reaction mixture was allowed to cool to room temperature, followed by the evaporation of toluene by distillation under high pressure. The resulting viscous, transparent yellow compound was washed in hexane five times (each 100 mL) and dried under vacuum for 48 h at room temperature. The polymer designations were poly(BZ-JD) and poly(BZ-JED). In order to indicate the particular structure of the Jeffamine^®^, molecular weight information was added; e.g., poly(BZ-JD230), poly(BZ-JED600), and poly(BZ-JED900) indicate molecular weights of 230, 600, and 900, respectively.

### 2.3. Crosslinking of PEG-Based Main Chain Benzoxazine Polymer

The polymers were dissolved in toluene (20 wt %). A film was cast over a levelled hydrophobic glass plate to prevent surface adhesion. Toluene was evaporated first at 60 °C for 12 h and then in a convection oven at 90 °C for 24 h. Cross-linking took place in a convection oven at 200 °C for 5 h and gave brown transparent polymer films. The temperature was gradually increased from 120 to 200 °C in intervals of 20 °C. The temperature steps were maintained for 2 h until a constant value was attained.

### 2.4. Characterization

#### 2.4.1. Proton Nuclear Magnetic Resonance (^1^H-NMR)

NMR spectra were recorded on a Bruker AV300 NMR spectrometer (Bruker Biospin GmbH, Karlsruhe, Germany) operating at a field of 7 Tesla (300 MHz) using a 5 mm ^1^H TXI probe and a sample temperature of 298 K. ^1^H spectra were recorded applying a 10 ms 90° pulse.

#### 2.4.2. Differential Scanning Calorimetry (DSC) 

DSC experiments were performed in a DSC1 (Starsystem) from Mettler Toledo, Gießen, Germany, using a nitrogen purge gas stream (60 mL/min) at a scan rate of 10 K/min. Heating and cooling scans were performed by initially heating the sample up to 150 °C and holding it at that temperature for 5 min in order to erase previous thermal history, if any, and the sample was then cooled down to 30 °C. Finally, a second heating scan up to 300 °C and a second cooling scan down to 30 °C was applied. The DSC thermograms presented here correspond to the second heating.

#### 2.4.3. Thermalgravimetric Analysis (TGA)

Thermogravimetric analysis (TGA) was used to investigate the onset degradation temperature and the measurements were obtained using a Netzsch TG209 F1 Iris instrument (NETZSCH-Gerätebau GmbH, Selb, Germany) under argon flow (20 mL min^−1^) from 25 to 900 °C at 10 K/min.

#### 2.4.4. Gas Separation Measurement 

Permeabilities of four pure gases (H_2_, N_2_, O_2_, CO_2_, and CH_4_) were measured by a pressure increase time-lag apparatus at 30 °C [20,21,22,23,24,25]. Permeability (*P*), diffusivity (*D*), solubility (*S*), and selectivity (*α_i/j_*) for gases *i* and *j* were determined under steady state conditions by the following equations:

(1) $$P=D\cdot S=\frac{V_{p}l\left(p_{p2}-p_{p1}\right)}{ART\Delta t\left[p_{f}-\frac{\left(p_{p2}+p_{p1}\right)}{2}\right]}$$

(2) $$D=\frac{l^{2}}{6\theta }$$

(3) $$\alpha_{i/j}=\frac{P_{i}}{P_{j}}=\frac{D_{i}S_{i}}{D_{j}S_{j}}$$

where *Vp* was the constant permeate volume, *R* was the gas constant, *l* was the film thickness, A was the effective area of the membrane, Δt was the time for the permeate pressure increase from *p_p_*_1_ to *p_p_*_2_, *p_f_* was the feed pressure, and *θ* was the time-lag. The solution–diffusion transport model [26] was applied for discussing the gas transport properties of polymeric membranes, and the selectivities of membranes for gas “*I*” relative to another gas “*j*” was the ratio of their permeabilities obtained from Equation (3).

## 3. Results and Discussion

### 3.1. Synthesis and Characterization of PEG-Based Main Chain Benzoxazine Polymer

Main-chain PEG type polybenzoxazines were synthesized via the typical polycondensation reaction of a Jeffamine^®^ with a 230, 600, and 900 g/mol molecular weight, Bisphenol-A, and paraformaldehyde [27,28,29,30,31], according to Scheme 1. 

The synthesis of PEG-based main-chain polybenzoxazine was dependent on the reaction of Jeffamine^®^, Bisphenol-A, and paraformaldehyde in the molar ratio of 1:1:4. The reaction conditions for the synthesis of poly(BZ-JD230) at the first place were examined. When the mixture was heated at 100 °C for 1 h without solvent (solvent-less method), insoluble bulky solid was obtained, suggesting the difficulties of applying the solvent-less method. Therefore, the solvent method was implemented. When the mixture was refluxed in the solvent (in our case toluene), the formation of gel was greatly decreased.

Different poly(ether diamine) chain length allows for design flexibility of the main chain- PEG type benzoxazine polymer, which consists of repeating units of a bifunctional benzoxazine structure bonded to a relatively large poly(ether diamine)s structure. Moreover, the Jeffamine^®^ and Bisphenol- A was a typical amino and phenolic ingredient for benzoxazine synthesis, respectively. Moreover, Bisphenol-A was common reagent for a wide range of commercial polymers, including polyesters, polycarbonates, and various epoxy resins. All of the synthesized polymers were soluble in organic solvents like chloroform, dioxane, dimethylformamide, dimethylacetamide, dimethyl sulfoxide, and toluene. Toluene was a chosen solvent for the synthesis due to good polymer solubility and minimal health concerns. The poly(ethylene glycol) backbone of Jeffamine^®^ might cause an enhancement of its solubility in water. As Jeffamine^®^ molecular weight increases solubility in water and alcohols increases. Poly(BZ-JD230) was insoluble in water, slight solubility in water was achieved with poly(BZ-JED600), and complete solubility was observed with poly(BZ-JED900). All of the polymers were viscous liquids at room temperature, the viscosity increasing with Jeffamine^®^ molecular weight. The molecular weight (*M*_w_) of the PEG-based main-chain polybenzoxazine was in the range of 8000–9000 g/mol with the broad polydispersity (3 to 4), showing that the molecular weight was not so high.

**Figure 1:**
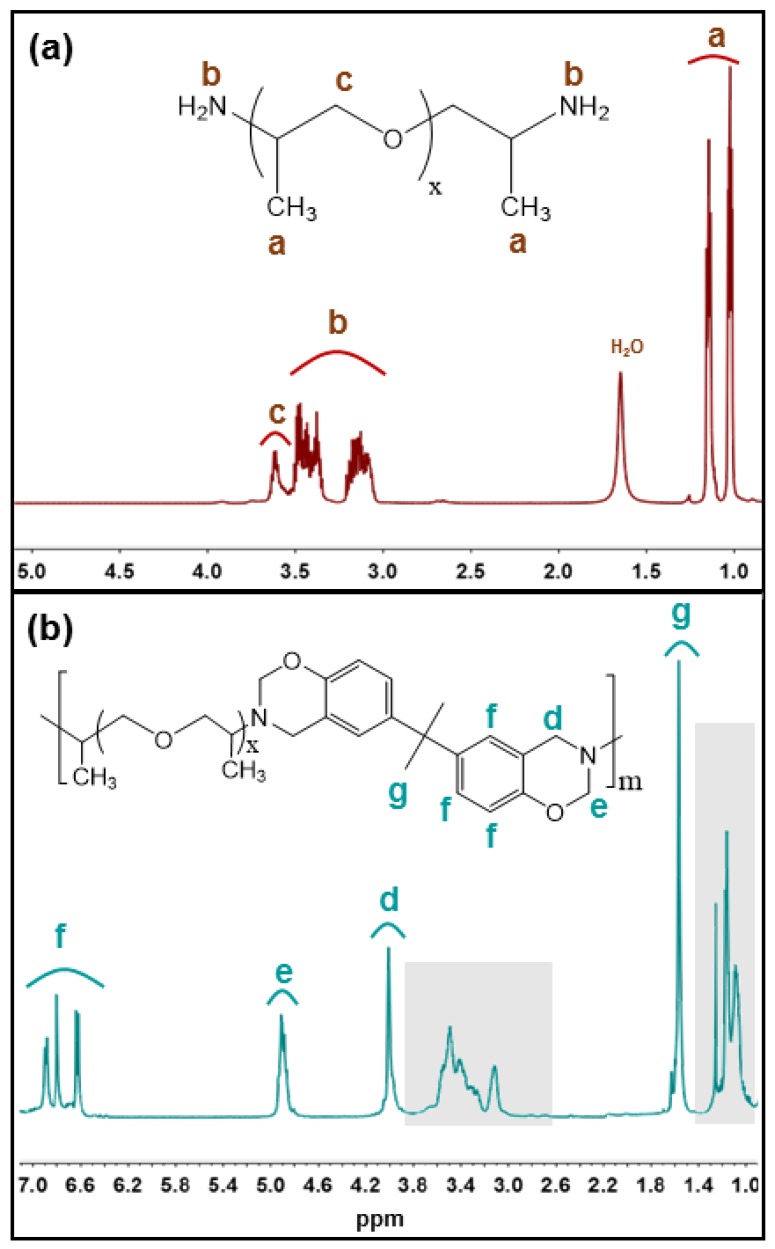
^1^H NMR spectra of (**a**) Jeffamine^®^ (JD-230) (**b**) Jeffamine^®^ benzoxazine polymer [Poly(BZ-JD230)].

The polymer (PEG-based polybenzoxazine) structure was confirmed by ^1^H-NMR spectra. Figure 1a shows the ^1^H NMR spectra of Jeffamine^®^ (JD230) and Figure 1b shows their corresponding benzoxazine polymer.

The peaks at 1.0–1.2 and 3.7 ppm correspond to the –CH_3_ and O–CH_2_– groups of the poly(propylene oxide) structure, respectively (Figure 1a). Benzoxazine structure was identified by the presence of the characteristic chemical shifts of the peak of equal integrated intensity due to the methylene groups in the cyclic benzoxazine structure. The peak at 4.0 ppm was due to the protons in C–CH_2_–Ph, and the peak at 4.9 ppm was due to the protons in O–CH_2_–N. The aromatic protons of the benzoxazine structure appear between 6.6 and 7.3 ppm (Figure 1b). The shaded region inside Figure 1b corresponds to the excess proton from Jeffamine^®^ (JD230) in the polymer. Polymers synthesized using other Jefamine^®^ (JED600 and JED900) reactants show similar ^1^H NMR spectra.

**Figure 2:**
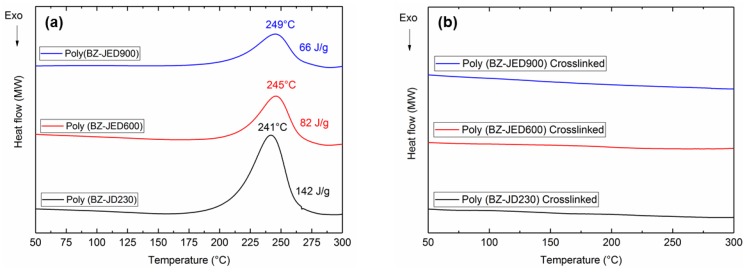
DSC thermograms (second heating cycle) of (**a**) uncrosslinked and (**b**) crosslinked PEG-based polybenzoxazine.

Differential scanning calorimetry (DSC) was used to study the thermal transition of both uncrosslinked and crosslinked PEG-based polybenzoxazine in the temperature range from 50 to 300 °C (Figure 2). The polymer film was first placed at 60 °C for 12 h to remove the solvent. In order to achieve further evaporation of the solvent before the beginning of cross-linking, samples were held at 90 °C for 24 h. Figure 2a depicts the DSC thermograms, illustrating the thermal behaviour of the uncrosslinked polymers. From the DSC results, a single exothermic peak is exhibited, which was due to the ring-opening polymerization (ROP) of the benzoxazine. All exothermic peaks were fairly broad, with total enthalpies of 142, 82, and 66 J/g and exothermic peaks at 241, 245, and 249 °C for poly(BZ-JD230), poly(BZ-JED600), and poly(BZ-JED900), respectively. As PEG length in the polymer was increased, the exothermic heat of polymerization attributed to benzoxazine ring-opening decreased due to a dilution effect [14]. The crosslinked polymers were also characterized by DSC and the results are shown in Figure 2b.

### 3.2. Characterization of Thermally Crosslinked PEG-Based Main Chain Benzoxazine Polymer 

**Figure 3:**
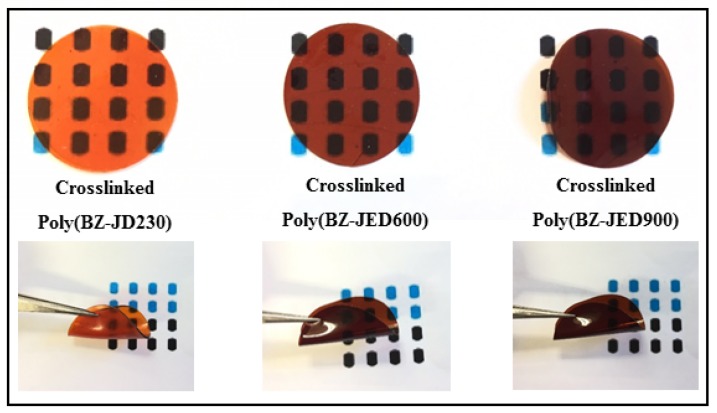
Physical appearance of crosslinked polybenzoxazine films.

The PEG-based polybenzoxazine materials were dissolved in toluene (20 wt %) and cast on a glass plate. The solvent was removed by drying at 60 °C for 12 h. After heat treatment, brown transparent polybenzoxazine films were obtained. The physical appearance of crosslinked films is shown in Figure 3. All crosslinked polybenzoxazine films were mechanically stable, tough, and ~70–80 µm thick.

**Figure 4:**
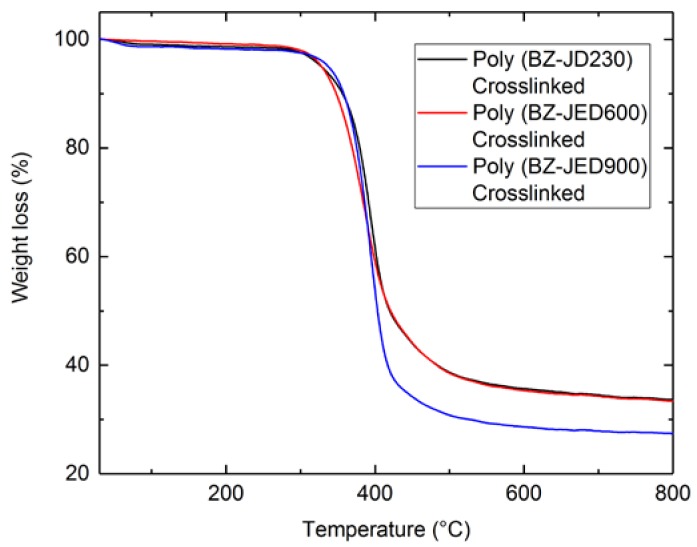
TGA of crosslinked PEG-based polybenzoxazine.

From the DSC data, it was confirmed that the crosslinking or ROP was observed between 200–270 °C for all PEG-based polybenzoxazine polymers. Therefore, poly(BZ-JD230), poly(BZ-JED600), and poly(BZ-JED900) films were crosslinked at 200 °C for 5 h. In order to check the thermal stability of crosslinked polymers, TGA was carried out under a nitrogen atmosphere and the results are shown in Figure 4. 

The onset temperature of degradation of the polymers increased with poly(ether diamine) chain length in Jefamine^®^. The values of 10 wt % weight reduction temperature, T_d10_, for poly(BZ-JD230), poly(BZ-JED600), and poly(BZ-JED900) were 340, 350, and 360 °C, respectively. It was possibly related to the C–N bond concentration at the polyether chain/benzoxazine linkage that could have been acting as the thermally weak link. As the polyether chain molecular weight decreases, the concentration of such links increases, which then might lead to the weakening of the thermal stability [32]. A similar case was reported for the main-chain-type polybenzoxazine, where the fluorinated aliphatic chain was connected to the amine group through a CH_2_ group. When this CH_2_ group was replaced with a phenyl group, the thermal stability was drastically enhanced [33]. The char yield at 800 °C increased as poly(ether diamine) length increased. Char yield for poly(BZ-JD230), poly(BZ-JED600), and poly(BZ-JED900) was 4.3, 4.0, and 3.3%, respectively (Table 2). This trend can be attributed to a higher density of benzoxazine cross-linking in the main chain. 

The crosslinked polymers were characterized by DSC and the results are shown in Figure 2b. There is a significant exothermic difference between non-crosslinked and crosslinked polymers (Figure 2a,b). The exotherm completely disappears after crosslinking, showing that ring opening of oxazine was completed for all crosslinked polymers. 

**Scheme 2:**
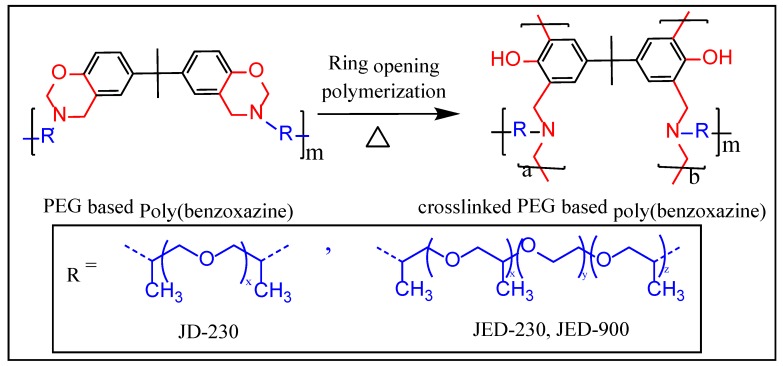
Predicted crosslinking mechanism of PEG-based polybenzoxazine.

The crosslinked structure was supported by insolubility in an organic solvent such as tetrahydrofuran, toluene, and chloroform. The possible structure of the PEG-based crosslinked polybenzoxazine is depicted in Scheme 2, according to reference [15].

### 3.3. Gas Transport Properties of Crosslinked PEG-Based Main Chain Benzoxazine Polymer

Free standing films of polymers (as shown in Figure 3) for gas separation could be prepared by thermal crosslinking and they were tough enough to allow a measurement of gas permeability, which was not possible for the precursor polymeric films. Single gas permeation for H_2_, N_2_, O_2_, CO_2_, and CH_4_ was carried out at 30 °C by the time-lag instrument on crosslinked polymer films. The data on the gas permeability coefficient and the values of ideal permselctivities are shown in Table 3. The order of the gas permeability was CO_2_ > O_2_ > CH_4_ > N_2_.

Polymers of heteroaromatic structures were characterized by a high level of selectivity but relatively low permeability in the gas separation process [34,35]. It was shown that the permeability coefficient of all gases in Poly(BZ-JD230) was lower than those of gases through the Poly(BZ-JED900) film. The reason for the high permeability of Poly(BZ-JED900) was due to the presence of PEG chains in the crosslinked polymeric membrane. The PEG group has a stronger interaction with a polar gas, such as CO_2_, than a nonpolar gas, e.g., N_2_. In that case, the polar gas solubility can be enhanced and the gas permeability increased, which facilitates the improvement of the total CO_2_/N_2_ and CO_2_/CH_4_ gas pair selectivity [36,37,38,39]. The permselectivity of CO_2_/N_2_ and CO_2_/CH_4_ for Poly(BZ-JED900) was found to be higher than those of the membrane Poly(BZ-JD230). 

**Figure 5:**
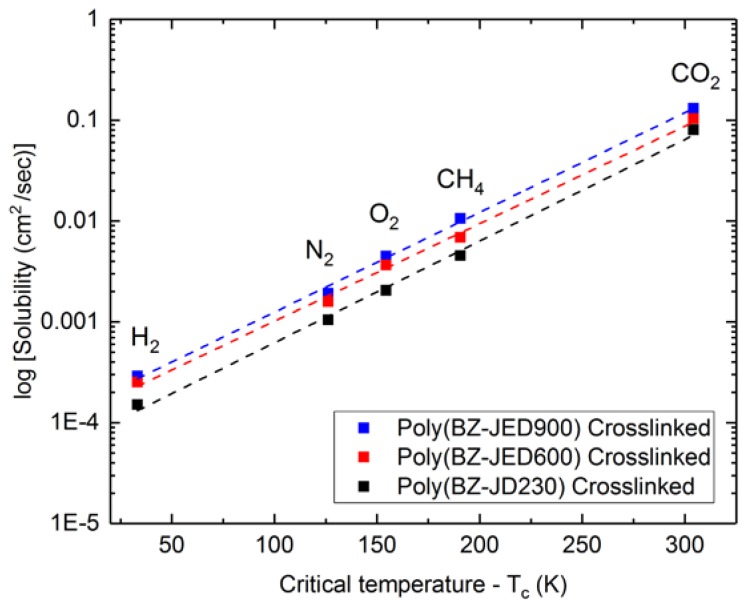
Dependence of gas solubility coefficients as a function of critical temperature (T_c_) of gas molecules for PEG-based polybenzoxazine.

Furthermore, the gas permeability tendency of crosslinked polymeric membranes was CO_2_ > O_2_ > CH_4_ > N_2_. This order was the same as for most polymeric rubbery membranes, which follows the solution-diffusion mechanism for the gas permeation. In this direction, the important key parameter determining the permeability coefficient was the critical temperature of the gases [40] [CO_2_ (304.2 K) > CH_4_ (190.6 K) > O_2_ (154.4 K) > N_2_ (126.2 K) > H_2_ (33.2 K), data according to [41]]. The gases with a higher critical temperature have a higher solubility coefficient and condensability. N_2_ with the lowest critical temperature would be expected to have the lowest permeability coefficient. Figure 5 clearly shows the dependence of the gas solubility coefficient as a function of the critical temperature of gas molecules in crosslinked PEG-based polybenzoxazine polymeric membranes. 

**Figure 6:**
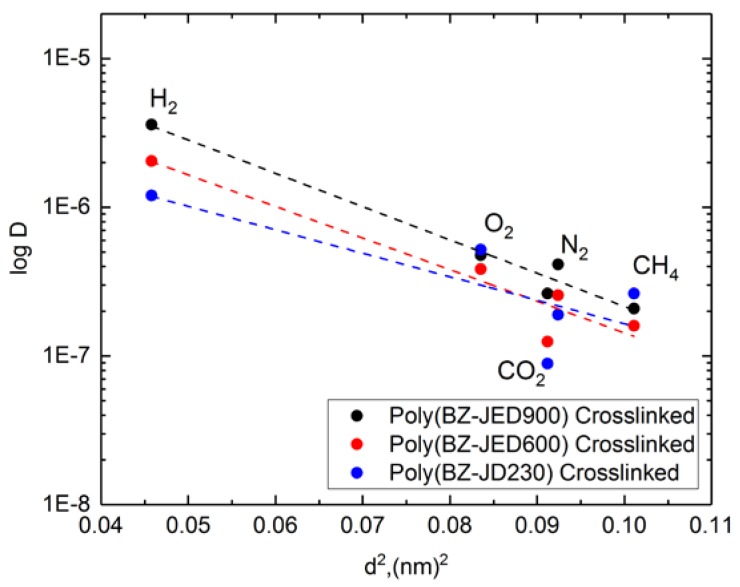
Dependence of gas diffusivity coefficients as a function of effective kinetic diameter of gas molecules for PEG-based polybenzoxazine.

Correlation analysis of the gas permeability parameters of the crosslinked PEG-based polybenzoxazine films was carried out using the Teplyakov method [42], which was based on the dependence of the gas diffusion coefficient, *D*, on the effective kinetic diameter of gas molecule, *d*, and the Equation can be given as:

(4) $$\log D=K_{1}-K_{2}\times d^{2}$$

where *K*_1_ and *K*_2_ were the constants. Usually, the coefficients of gas diffusion through a polymer depend linearly on the diameter of the gas molecules. Figure 6 shows the correlation dependence of diffusion coefficients on the effective kinetic diameter of gas molecules. The points corresponding to the gas diffusivity (log *D*) values as a function of effective kinetic diameter (*d*) for the polybenzoxazine films display a distinct deviation from linearity. The deviation from linearity could indicate the presence of some specifics of the polymer structure and excess free volume in the polymer. The order of effective kinetic diameter was CH_4_ (0.318 nm) > N_2_ (0.304 nm) > CO_2_ (0.302) > O_2_ (0.289 nm) > H_2_ (0.214 nm) [43]; however, the smallest kinetic diameter of H_2_ leads to a high diffusion coefficient that makes it more permeable than all other gases except CO_2_.

**Figure 7:**
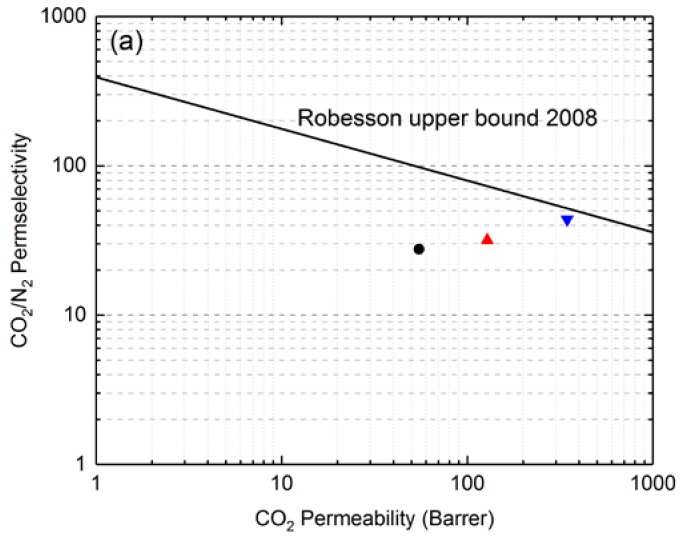

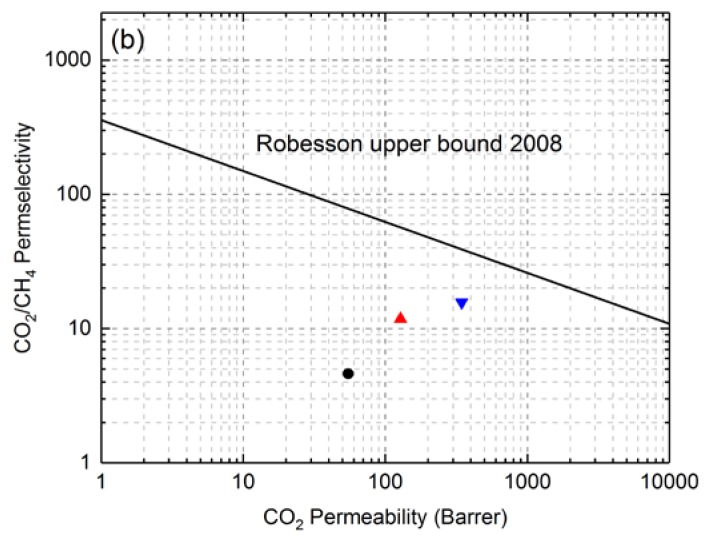
Robeson plot for (**a**) CO_2_/N_2_ and (**b**) CO_2_/CH_4_ gas pair showing data for PEG-based polybenzoxazine films measured at 30°C. [●—Poly(BZ-JD230), ▲—Poly(BZ-JED600), and ▼—Poly(BZ-JED900)].

In order to see the performance of crosslinked PEG-based polybenzoxazine polymeric materials among known gas-separation materials, their transport properties were plotted on a Robeson plot (Figure 7). Robeson analyzed data on permeability and selectivity for a large number of glassy and rubbery polymers and established the position of the upper bound line for several gas pairs in 1991; before redefining them in 2008 [3]. Figure 7 shows the upper bound line for membrane separation of the CO_2_/N_2_ and CO_2_/CH_4_ gas pairs. It was observed that polybenzoxazine polymeric films were located near the Robeson upper bound lines and that the performance of the poly(BZ-JED900) film was better than that of the poly(BZ-JD230) film in the separation of both the CO_2_/N_2_ and CO_2_/N_2_ gas pairs.

## 4. Conclusions

The poly(ethylene glycol)-based polybenzoxazine precursor containing a cyclic benzoxazine group in the backbone was synthesized successfully by a polycondensation reaction between Bisphenol-A, paraformaldehyde, and poly(ether diamine) known as Jeffamine^®^. The precursor polymer solutions were cast on a glass plate and crosslinked via thermally activated ring opening polymerization of the cyclic benzoxazine structure. The flexible and mechanically stable polymer films were obtained without using any external initiators, accelerators, catalysts, or reactive diluents. These crosslinked polymers films were characterized by DSC and TGA. Gas permeation of the crosslinked films was measured using single gas H_2_, O_2_, N_2_, CO_2_, and CH_4_. The gas permeability and permselectivity (CO_2_/N_2_ and CO_2_/CH_4_ gas pairs) of polybenzoxazine polymers increases as the PEG chain length increases on polybenzoxazine. Correlation analysis of gas diffusion showed a linearity in crosslinked films. The position of these crosslinked PEG-based polybenzoxazine films on the Robeson plot was close to the upper bound line for the CO_2_/N_2_ and CO_2_/CH_4_ gas pairs. The unique feature of this class of materials provides the possibility of preparing efficient thin film composite membranes for various gas and liquid membrane separation applications. 

## Figures and Tables

**Table 1:** Chemical structure, composition, and molecular weights of Jeffamine^®^ used for polymer synthesis.

Commercial Name	Code	Chain Architecture		Molecular Weight (g/mol)
		x		
Jeffamine^®^ D-230	JD230	~2.5		230
Jefamine^®^ D structure	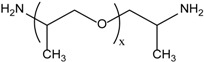			
Jeffamine^®^ ED-600	JED600	y	x + z	600
		~9	~3.6	
Jeffamine^®^ ED-900	JED900	~12.5	~6	900
Jefamine^®^ ED structure	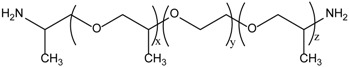			

**Table 2:** Thermal degradation of PEG-based polybenzoxazine materials.

Polymer	T_d10_ (°C)	Onset Temperature (°C)	Char Yield (%)
Poly(BZ-JD230)	340	370	4.3
Poly(BZ-JED600)	350	380	4.0
Poly(BZ-JED900)	360	390	3.3

**Table 3:** Gas separation performance of PEG-based polybenzoxazine.

Polymers	Permeability Coefficient (Barrer ^1^)				Permselectivity		
	O_2_	N_2_	CO_2_	CH_4_	O_2_/N_2_	CO_2_/N_2_	CO_2_/CH_4_
Poly(BZ-JD230)	11	2	55	11	5.4	27.6	4.6
Poly(BZ-JED600)	14	4	128	12	3.4	31.8	11.8
Poly(BZ-JED900)	21	8	345	22	2.7	43.5	15.7

^1^ $\mathrm{Barrer}=1*10^{-10}\cdot \frac{{\mathrm{cm}}_{\mathrm{STP}.}^{3}\mathrm{cm}}{{\mathrm{cm}}^{3}.\sec.\mathrm{cmHg}}$.
